# Unplanned Readmissions Due to Post-acute Myocardial Infarction Complications: Insights From the Nationwide Readmission Database (2016-2020)

**DOI:** 10.7759/cureus.108815

**Published:** 2026-05-13

**Authors:** Mohammad Hamza Bin Abdul Malik, Muhammad Arham, Hanzala Jehangir, Ayesha Ihsan, Adil Ahmed, Muhammad Ans Bin Abdul Malik, Hareem Saleem, Muhammad Salaar Riaz, Muhammad Awais Bin Abdul Malik, Muhammad Faizan Ali, Faizan Ahmed, Sherif Eltawansy, Ahmad Elashery

**Affiliations:** 1 Internal Medicine, Nassau University Medical Center, New York City, USA; 2 Research, Sheikh Zayed Medical College, Rahim Yar Khan, PAK; 3 Internal Medicine, Rutgers-Trinitas Regional Medical Center, Elizabeth, USA; 4 Internal Medicine, University Medical and Dental College, Faisalabad, PAK; 5 Medicine and Surgery, Northwest School of Medicine, Peshawar, PAK; 6 Internal Medicine, Sheikh Zayed Medical College Hospital, Rahim Yar Khan, PAK; 7 Internal Medicine, Memorial Healthcare System, Hollywood, USA; 8 Internal Medicine, Advent Health Orlando, Orlando, USA; 9 Internal Medicine, Jinnah Postgraduate Medical Center, Karachi, PAK; 10 Internal Medicine, Jersey Shore University Medical Center, Neptune, USA; 11 Cardiology, Charleston Area Medical Center, South Charleston, USA

**Keywords:** complications’, heart failure patients, myocardial infarction, patient readmission, post myocardial infarction (mi)

## Abstract

Introduction

Acute myocardial infarction (MI) readmissions within 30 days (30-dr) are affecting patient outcomes and healthcare costs. This study analyzed trends in 30-dr for patients discharged after an acute MI.

Methods

We analyzed the 2016-2020 Nationwide Readmission Database for patients aged 18 years or older with an initial admission for acute MI who were readmitted within 30 days. Variables were identified using ICD-10 codes. The primary outcome was trends in 30-dr; secondary outcomes included trends in complications, mortality, length of stay (LOS), and healthcare costs. Multivariate and descriptive bivariate analyses were conducted, with p-values <0.05 considered statistically significant.

Results

Among 2,572,790 acute MI index admissions, 221,910 (8.6%) were readmitted within 30 days, with a significant decline in readmission risk over the study period (p < 0.001). Mean age was 66.9 ± 13.5 years. In-hospital mortality decreased over time (OR 0.92, 95% CI 0.88-0.96; p trend < 0.01). During index admissions, vasopressor use and acute kidney injury increased, while periprocedural bleeding declined (OR 0.38, 95% CI 0.33-0.43). In multivariable Cox regression, 30-day readmission risk declined from 2017 to 2019 (HR 0.93 to 0.87) with a slight increase in 2020 (HR 0.94). Higher age (HR 1.01) and comorbidity burden (HR 1.07) were associated with increased risk, while male sex was protective (HR 0.92 [0.90-0.94]). Periprocedural circulatory complications (OR 0.30, 95% CI 0.17-0.51) and bleeding (OR as low as 0.07, 95% CI 0.03-0.17) declined, while post-procedural anemia (OR 1.16, 95% CI 1.06-1.26) and non-inflammatory pericardial effusion (OR 1.62, 95% CI 1.34-1.97) increased.

Conclusion

30-dr after acute MI declined over time, but remains driven by increasing comorbidity burden and evolving procedural complication profiles, underscoring the need for targeted risk stratification and post-discharge care. This study highlights relevant data to inform targeted interventions to reduce readmissions and complications.

## Introduction

In the United States, an estimated 805,000 individuals suffer an acute myocardial infarction (MI) annually, translating to one heart attack every 40 seconds [1]. The Task Force for the Universal Definition of Myocardial Infarction (UDMI) defines MI as an injury indicated by elevated cardiac biomarkers, specifically cTn values above the 99th percentile of the normal range. The injury is considered acute if there is a rise and/or fall in cTn levels, along with evidence of acute myocardial ischemia [2,3]. Despite therapeutic strides, such as an 86% increase in reperfusion rates over the past decade (Dasari et al.), 30-day readmissions (30-dR) post-acute MI remain a persistent challenge in our care continuum [4]. 30-dR is an established performance metric for hospitals, with approximately 20% of Medicare beneficiaries readmitted within 30 days after acute MI [5,6]. Efforts to mitigate this include the Centers for Medicare & Medicaid Services (CMS) public reporting of 30-day risk-adjusted re-admission rates [7,8].

A recent meta-analysis by Wang et al. [9] identified acute MI, angina, acute ischemic heart disease, and heart failure (HF) as leading causes of 30-day re-admissions. The Medicare Standard Analytic and Denominator Files (2007-2009) recorded 548,834 hospitalizations for acute MI [10]. Studies utilizing databases such as the Nationwide Readmissions Database (NRD) and Medicare Fee-for-Service have found that approximately 19% of acute MI cases result in unplanned readmissions, leading to healthcare costs exceeding $17 billion [10-12]. The re-admission rate of patients who underwent coronary artery bypass grafting (CABG) post-acute MI rose to 25% [13].

Hospital re-admissions are critical indicators of healthcare quality. Efforts to mitigate them include the Centers for Medicare & Medicaid Services (CMS) public reporting of 30-day risk-adjusted re-admission rates [10,11]. However, 30% of re-admissions are still preventable [12]. Studies like those by Rymer et al. [14] have cataloged risk factors, including a previous history of acute MI, HF, major vascular bleeding, and differences in reperfusion strategies. Studies have shown that in-hospital events, such as tachyarrhythmias (VT/AF), are associated with higher re-admission rates, and age at admission is an independent predictor [15]. Recently, the Elixhauser Comorbidity Index has demonstrated excellent predictive ability for in-hospital mortality and 30-dR (30-day), underscoring the need for a more pragmatic index value [16,17]. The impact of length of stay on 30-day readmission rates is also being examined in studies, such as those by Jang et al. [18]. In this context, a prior study using the NRD database demonstrated that AMI survivors impose a substantial burden on U.S. healthcare resources; however, its scope was limited to 2013 data, whereas we provide updated coverage from 2016 to 2020 [11].

Given these unmet needs and existing disparities, we analyzed the 2016-2020 Nationwide Readmission Database for patients aged 18 years or older who had an initial admission for acute MI and experienced a 30-day readmission. By accurately characterizing index hospitalizations and 30-day readmissions, we sought to enhance the existing frameworks governing pre- and post-discharge care protocols.

## Materials and methods

Data source

The study used data from the Nationwide Readmissions Database (NRD) for 2016-2020, administered by the Agency for Healthcare Research and Quality (AHRQ) through the Healthcare Cost and Utilization Project (HCUP). The NRD is the largest all-payer database for hospital readmissions in the United States, providing a nationally representative sample. Data are coded using the ICD-10-CM and ICD-10-PCS systems, capturing comprehensive information on patient demographics, diagnoses, and procedures.

Inclusion and exclusion criteria

This study included patients diagnosed with acute MI who were discharged between 2016 and 2020 and had a 30-day follow-up. Acute MI was defined as a composite of Non-ST-segment Elevation Myocardial Infarction (NSTEMI), identified by ICD-10 code I21.4, and ST-segment Elevation Myocardial Infarction (STEMI), identified by ICD-10 codes I21.0, I21.01, I21.02, I21.09, I21.1, I21.11, I21.19, I21.2, I21.21, I21.29, and I21.3. Patients who died during their inpatient stay were excluded, as indicated by the DISPUNIFORM variable (which records discharge disposition, including in-hospital mortality). Planned readmissions within 30 days, identified using the ELECTIVE variable, were also excluded. Furthermore, patients discharged in December were excluded from the analysis because they would not have had a complete 30-day follow-up due to the data's annualized nature.

Study endpoints and confounders

The primary outcome was 30-day readmission, defined as unplanned rehospitalization within 30 days of discharge. Secondary outcomes included complications within 28 days of discharge, including congestive heart failure (CHF) (ICD-10 codes I50, I50.1, I50.2, I50.20, I50.21, I50.23, I50.3, I50.30, I50.31, I50.33, I50.40, I50.41, I50.43, I50.8, I50.81, I50.810, I50.811, I50.813, I50.814, I50.82, I50.83, I50.84, I50.89, I50.9), stroke (ICD-10 codes I60, I61, I62, I63, I65, I66, I67, I68, I69), gastrointestinal bleeding (ICD-10 codes K92, K92.0, K92.1, K92.2, K92.8, K92.81, K92.89, K92.9), arrhythmia (ICD-10 codes I49 series), and recurrent myocardial infarction (ICD-10 codes I21 and I22 series). Several potential confounders were identified and controlled for to account for variables that may influence both primary and secondary outcomes. These included demographic, clinical, and treatment-related factors. Smoking status was recorded using ICD-10 code Z72.0, while F10.19 captured a history of alcohol abuse. Cardiovascular risk factors, such as hyperlipidemia (E78.5), diabetes (E11.9), and hypertension (I10), were included, along with chronic kidney disease (CKD) identified by codes N28.9, N18.9, and N19. Additional cardiovascular conditions such as atherosclerotic heart disease (I25.10), old myocardial infarction (I25.2), and a history of coronary interventions, including coronary stent placement (Z98.61) and coronary artery bypass graft (Z95.1), were also considered. These confounders were carefully controlled to minimize their impact on the observed relationships between the primary and secondary outcomes and the exposure variables.

Statistical analysis

**Figure 1:**
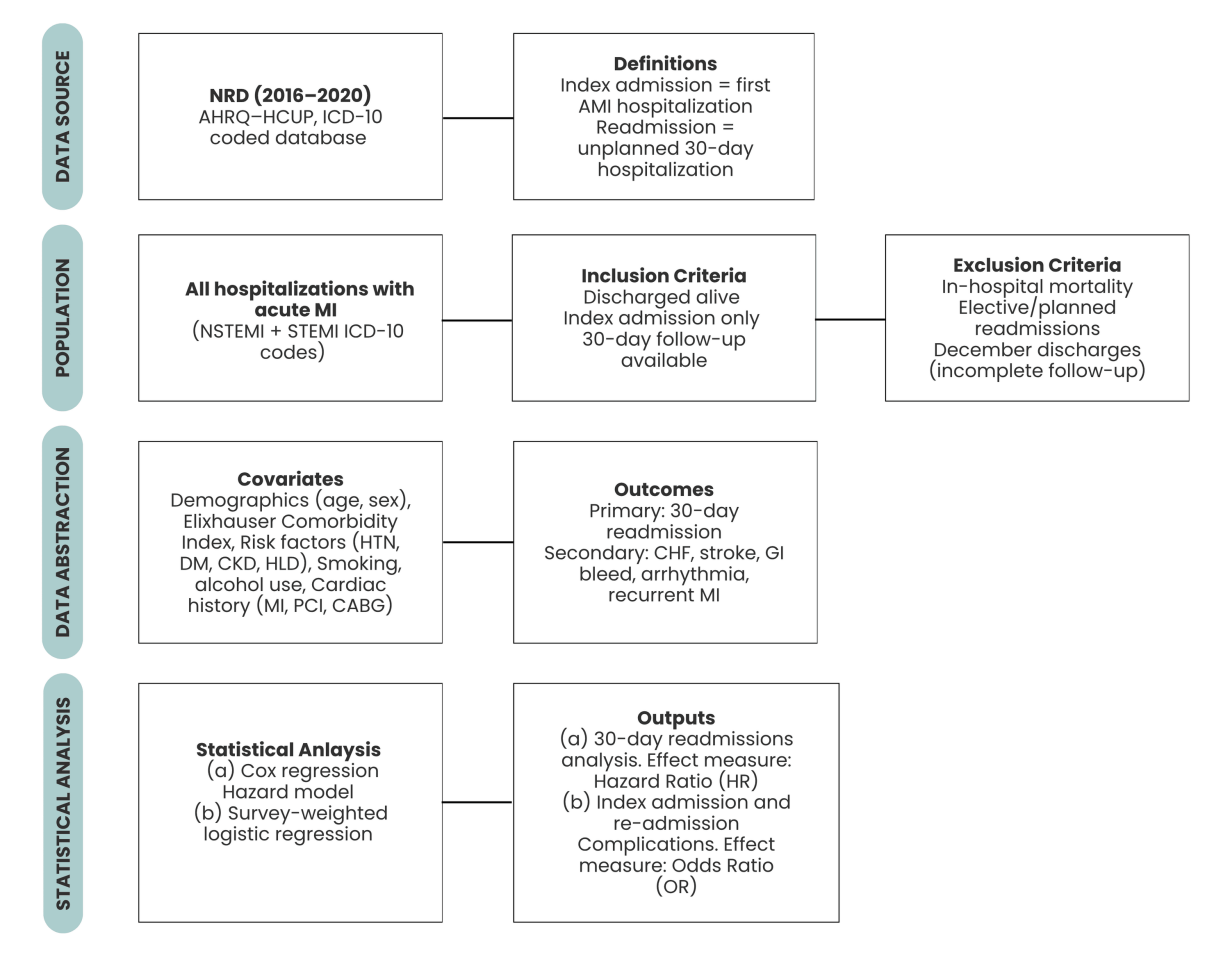
Study design and analytic framework for 30-day readmission and complication analysis using the Nationwide Readmissions Database (2016–2020) NRD: Nationwide Readmissions Database; AHRQ: Agency for Healthcare Research and Quality; HCUP: Healthcare Cost and Utilization Project; ICD: International Classification of Diseases; NSTEMI: Non-ST-segment Elevation Myocardial Infarction; CKD: Chronic kidney disease; HLD: Hyperlipidemia; HTN: Hypertension; DM: Diabetes mellitus; CABG: Coronary artery bypass grafting; MI: Myocardial infarction; PCI: Percutaneous coronary intervention; CHF: Congestive heart failure.

Continuous variables were reported as mean (SD) or median (IQR), and categorical variables as frequencies and percentages. Survival analysis was performed using time from discharge to readmission as the time-to-event variable, with censoring at 30 days for patients not readmitted. The primary outcome of 30-day readmission was evaluated using univariable and multivariable Cox proportional hazards regression models, generating hazard ratios (HRs). Covariates were selected if p < 0.2 in univariable analysis and included in multivariable models. The proportional hazards assumption was assessed using Schoenfeld residuals. Binary outcomes, including index admission and readmission-related complications, were analyzed using logistic regression models to estimate odds ratios (ORs). Temporal trends in outcomes across study years (2016-2020) were assessed using regression models with calendar year as a continuous variable (linear regression for continuous outcomes and logistic regression for binary outcomes), adjusted for age, sex, and Elixhauser Comorbidity Index. Marginal standardization was applied to derive adjusted estimates for population-level interpretation. Multiple comparisons were controlled using the Hochberg procedure. HCUP complex survey design was incorporated using sampling weights, strata, and primary sampling units to generate nationally representative estimates (Figure 1). All analyses were conducted using Stata version 16.1 (StataCorp, College Station, Texas, USA). Statistical significance was defined as a two-sided p < 0.05.

## Results

Baseline characteristics

Between 2016 and 2020, 2,572,790 patients were hospitalized for acute MI. The number of admissions increased yearly, peaking at 538,365 in 2019, before declining to 471,292 in 2020. Of the total admissions, 2,445,806 patients (95%) were discharged alive. During the same period, 221,910 patients were readmitted within 30 days of discharge. Readmissions steadily decreased over the years, from 47,790 in 2016 to 36,789 in 2020 (Table 1).

**Table 1:** Admission details of the patients with unplanned readmissions due to post-acute myocardial infarction (MI).

Patient Admission Details						
Patient admission details (n)	2016-2020	Stratified by calendar year				
		2016	2017	2018	2019	2020
Index Admissions	2,572,790	507,608	526,481	529,044	538,365	471,292
Index Admissions, Discharged Alive	2,445,806	481,621	500,663	503,463	512,886	447,173
Readmissions	221,910	47,790	46,051	45,717	45,561	36,789

The baseline characteristics of the patients showed a mean age of 66.9 years, with only slight variations across the study period. The average Elixhauser Comorbidity Index increased from 3.8 in 2016 to 4.0 in 2020, indicating a rising burden of comorbid conditions. Patient admissions rose consistently each year, reaching a peak of 538,365 in 2019 before dropping to 471,292 in 2020. The patient population was predominantly male, accounting for 1,613,139 (62.7%) of admissions. Insurance coverage remained consistent throughout the study period, with Medicare covering approximately 1,543,674 (60%) of patients, followed by private insurance at 676,643 (26.3%), Medicaid at 239,269 (9%), and self-pay accounting for the remainder. Most patients were from urban areas (85%), with large metropolitan hospitals accounting for 1,829,253 (70%) of admissions. These hospitals were primarily teaching institutions with large bed sizes (>500 beds), handling 1,463,917 (56.9%) of cases, while medium-sized 730,672 (28.4%) and small hospitals 375,627 (14.6%) managed the rest. Socioeconomic diversity was evident, with 776,982 (30.2%) of patients from the lowest income quartile. Pre-existing conditions were common among the cohort, with 949,359 (36.9%) of patients having cardiac arrhythmias, 612,324 (23.8%) diagnosed with renal failure, and 1,021,397 (39.7%) living with diabetes. Additionally, 216,114 (8.4%) of patients had a do-not-resuscitate (DNR) order documented. Baseline patient characteristics of the patients are presented in Table 2.

**Table 2:** Baseline patient characteristics of the patients with unplanned readmissions due to post-acute myocardial infarction (MI).

Baseline Patient Characteristics						
Patient Characteristics	2016-2020	Stratified by calendar year				
		2016	2017	2018	2019	2020
Patient (n)	2,572,790	507,608	526,481	529,044	538,365	471,292
Age in years at admission	66.9 ± 13.5	67.0 ± 13.5	67.0 ± 13.6	66.9 ± 13.5	66.9 ± 13.5	66.5 ± 13.4
Elixhauser Comorbidity Summary	3.9 ± 2.3	3.8 ± 2.2	3.9 ± 2.3	4.0 ± 2.3	4.0 ± 2.3	4.0 ± 2.3
Indicator of sex						
Male	1,613,139	316,240	326,945	331,181	337,555	301,627
Female	959,650	191,368	199,536	197,862	200,810	169,665
Insurance Carrier, cleaned						
Medicare	1,543,674	307,103	319,574	318,484	321,942	275,235
Medicaid	239,269	45,177	48,436	49,201	50,068	46,658
Private insurance	676,643	133,501	136,359	137,551	142,128	127,249
Self-pay	113,202	21,827	22,112	23,806	23,688	22,151
Median household income national quartile for patient ZIP Code						
0-25th percentile	776,982	154,313	160,050	157,655	163,125	140,916
26th to 50th percentile (median)	740,963	139,085	153,732	156,067	150,204	142,330
51st to 75th percentile	617,469	124,872	124,776	127,499	132,438	107,926
76th to 100th percentile	437,374	89,339	87,396	88,350	92,599	80,120
Bed size of hospital						
Small	375,627	64,466	72,128	79,356	85,062	75,407
Medium	730,672	141,115	155,312	150,777	152,896	131,490
Large	1,463,917	302,027	299,568	298,909	300,408	264,395
Hospital urban-rural designation						
Large metropolitan areas with at least 1 million residents	1,286,395	254,312	266,926	265,580	268,106	231,404
Small metropolitan areas with less than 1 million residents	1,111,445	218,779	223,228	227,488	234,727	207,840
Micropolitan areas	154,367	30,456	32,115	31,742	31,764	28,749
Not metropolitan or micropolitan (non-urban residual)	20,582	4,568	4,212	4,232	3,769	3,299
Teaching status of urban hospitals						
Metropolitan non-teaching	568,586	140,607	125,302	112,157	100,136	89,074
Metropolitan teaching	1,829,253	331,976	364,851	380,911	402,697	350,170
Non-metropolitan hospital	174,949	34,517	36,327	35,974	35,532	32,048
Patient Location: NCHS Urban-Rural Code						
Central counties of metro areas of >=1 million population	560,868	113,197	117,932	116,918	115,748	97,086
Fringe counties of metro areas of >=1 million population	635,479	123,349	130,567	130,673	133,515	118,294
Counties in metro areas of 250,000-999,999 population	576,304	113,704	116,879	117,447	121,132	107,455
Counties in metro areas of 50,000-249,999 population	283,006	55,329	56,860	58,723	59,220	52,313
Micropolitan counties	283,006	55,329	57,913	57,665	59,220	52,313
Not metropolitan or micropolitan counties	234,123	46,700	46,330	47,084	48,991	43,830
Admission day is a weekend						
Admitted Monday-Friday	1,875,563	369,031	383,278	385,144	391,930	344,986
Admitted Saturday-Sunday	697,226	138,577	143,203	143,899	146,435	126,306
Cardiac Arrhythmias						
Absent	1,623,430	325,377	336,421	334,355	335,940	292,201
Present	949,359	182,231	190,060	194,688	202,425	179,091
Renal Failure						
Absent	1,960,465	389,335	401,705	402,602	407,004	359,125
Present	612,324	118,273	124,776	126,441	131,361	112,167
Diabetes						
Absent	1,551,392	312,687	317,995	317,426	322,481	282,304
Present	1,021,397	194,921	208,486	211,617	215,884	188,988
Do Not Resuscitate Order						
Absent	2,356,675	466,999	482,257	484,075	491,527	430,761
Present	216,114	40,609	44,224	44,968	46,838	40,531

Index admission complications

Index Admission Characteristics

The inpatient admission mortality rate obtained from the index first admission dropped consistently through the study years, with an odds ratio (OR) of 0.92 (confidence interval [CI], 0.88-0.96) by 2020, representing a decrease of 0.08 OR from 2016 (p trend < 0.01), after using logistic Cox-regression. Meanwhile, the index admission LOS rate was persistent through the study years, with an overall average of 4.64 days (p trend = 0.25). Table 3 presents the analysis of mortality, length of stay, and hospitalization costs.

**Table 3:** Index admission characteristics of the patients with unplanned readmissions due to post-acute myocardial infarction (MI)

Index admission characteristics									
Characteristic	Mortality			Length of stay			Total Cost		
	Odds Ratio	95% CI	p-value	Regression Coefficient	95% CI	p-value	Regression Coefficient	95% CI	p-value
Calendar year									
2016	1.00			0.00			0.00		
2017	0.91	[0.87 - 0.96]	0.000	-0.13	[-0.22 --0.04]	0.007	342.92	[-277.16 -963.01]	0.278
2018	0.88	[0.84 - 0.93]	0.000	-0.17	[-0.26 --0.08]	0.000	889.81	[277.47 -1502.14]	0.004
2019	0.84	[0.80 - 0.88]	0.000	-0.22	[-0.31 - -0.12]	0.000	2038.82	[1359.25 -2718.40]	0.000
2020	0.92	[0.88 - 0.96]	0.000	-0.35	[-0.45 --0.26]	0.000	3916.99	[3160.57 -4673.40]	0.000
Age in years at admission	1.00	[1.00 - 1.00]	0.000	0.00	[0.00 - 0.00]	0.000	-144.83	[-151.44 - -138.23]	0.000
Elixhauser Comorbidity Summary	1.23	[1.23 - 1.24]	0.000	1.03	[1.01 - 1.05]	0.000	3436.45	[3345.94 - 3526.96]	0.000
Indicator of sex	0.86	[0.84 - 0.87]	0.000	-0.37	[-0.39 - -0.35]	0.000	-4106.29	[-4220.45 - -3992.13]	0.000
Insurance Carrier, cleaned									
Medicare	1.00			0.00			0.00		
Medicaid	0.96	[0.92 - 1.00]	0.041	0.37	[0.32 - 0.43]	0.000	365.60	[87.07 - 644.13]	0.010
Private insurance	0.85	[0.82 - 0.88]	0.000	0.16	[0.13 - 0.19]	0.000	1597.81	[1420.96 - 1774.66]	0.000
Self-pay	1.29	[1.22 - 1.37]	0.000	0.04	[-0.01 - 0.09]	0.141	-866.45	[-1178.33 - -554.57]	0.000
Median household income national quartile for patient ZIP Code									
0-25th percentile	1.00			0.00			0.00		
26th to 50th percentile (median)	0.96	[0.94 - 0.99]	0.008	-0.09	[-0.12 - -0.05]	0.000	1777.63	[1555.13 - 2000.14]	0.000
51st to 75th percentile	0.90	[0.87 - 0.93]	0.000	-0.16	[-0.21 - -0.12]	0.000	3173.42	[2874.69 - 3472.15]	0.000
76th to 100th percentile	0.89	[0.86 - 0.93]	0.000	-0.22	[-0.29 - -0.16]	0.000	5600.45	[5122.64 - 6078.27]	0.000
Bed size of hospital									
Small	1.00			0.00			0.00		
Medium	1.11	[1.06 - 1.17]	0.000	0.43	[0.35 - 0.51]	0.000	2285.70	[1640.20 - 2931.19]	0.000
Large	1.26	[1.20 - 1.32]	0.000	1.10	[1.02 - 1.17]	0.000	5984.22	[5391.09 - 6577.35]	0.000
Hospital urban-rural designation									
Large metropolitan areas with at least 1 million residents	1.00			0.00			0.00		
Small metropolitan areas with less than 1 million residents	0.96	[0.91 - 1.01]	0.132	-0.58	[-0.68 - -0.49]	0.000	-3114.48	[-3794.86 - -2434.10]	0.000
Micropolitan areas	0.87	[0.80 - 0.94]	0.000	-1.25	[-1.39 - -1.11]	0.000	-5169.69	[-6052.81 - -4286.58]	0.000
Not metropolitan or micropolitan (non-urban residual)	1.28	[1.12 - 1.46]	0.000	-0.67	[-0.89 - -0.44]	0.000	-5205.54	[-6597.83 - -3813.25]	0.000
Teaching status of urban hospitals									
Metropolitan non-teaching	1.00			0.00			0.00		
Metropolitan teaching	1.05	[1.02 - 1.09]	0.002	0.64	[0.58 - 0.70]	0.000	3194.07	[2758.88 - 3629.25]	0.000
Non-metropolitan hospital	1.00			0.00			0.00		
Patient Location: NCHS Urban-Rural Code									
Central counties of metro areas of >=1 million population	1.00			0.00			0.00		
Fringe counties of metro areas of >=1 million population	0.95	[0.92 - 0.98]	0.004	0.13	[0.06 - 0.20]	0.000	-1980.06	[-2501.90 - -1458.23]	0.000
Counties in metro areas of 250,000-999,999 population	1.03	[0.97 - 1.09]	0.375	0.39	[0.29 - 0.49]	0.000	810.95	[141.61 - 1480.29]	0.018
Counties in metro areas of 50,000-249,999 population	0.99	[0.93 - 1.06]	0.839	0.39	[0.28 - 0.49]	0.000	2025.60	[1328.90 - 2722.31]	0.000
Micropolitan counties	1.00	[0.95 - 1.06]	0.926	0.43	[0.33 - 0.53]	0.000	1383.82	[745.17 - 2022.46]	0.000
Not metropolitan or micropolitan counties	0.98	[0.92 - 1.05]	0.621	0.43	[0.33 - 0.54]	0.000	1599.83	[944.27 - 2255.40]	0.000

Procedural Complications

The use of in-hospital CPR rates showed a significant increase in 2020, with odds rising to 1.12 [95% CI: 1.04-1.21] compared to the earlier years in the study period. The use of defibrillation remained stable, with no significant changes in odds across the study period. Mechanical ventilation and extracorporeal membrane oxygenation (ECMO) were associated with increased risks, although these differences were not statistically significant. The use of vasopressors increased significantly over the study period, with odds rising from 1.00 in 2016 to 2.27 [95% CI: 1.82-2.83] in 2020 (p < 0.001), reflecting more intensive hemodynamic support in later years. Central line placements and blood transfusions were consistently utilized, maintaining stable odds throughout the study period.

Post-Procedural Complications

The risk of periprocedural bleeding steadily declined from 2016 to 2020, with OR improving from 0.40 [95% CI: 0.35-0.45] in 2016 to 0.38 [95% CI: 0.33-0.43] in 2020 (p < 0.001). Post-procedural anemia remained essentially unchanged, with OR consistently around 1.0 across all years, indicating no significant shifts in risk (p > 0.05). In contrast, the incidence of acute kidney injury increased over time, with OR reaching 1.10 [95% CI: 1.06-1.15] in 2020 (p < 0.01). Post-procedural respiratory complications also demonstrated a marked improvement over time, with OR decreasing to 0.53 [95% CI: 0.37-0.76] in 2020 (p < 0.001) with post-procedural respiratory failure demonstrating significant improvement, with OR dropping from 0.76 [95% CI: 0.64-0.91] in 2016 to 0.55 [95% CI: 0.45-0.68] in 2020 (p < 0.001).

Cardiac-Specific Complications

Non-inflammatory pericardial effusion exhibited a significant increase over time, with OR rising from 2018 (OR: 1.20, 95% CI: 1.09-1.32, p < 0.001) and peaking in 2020 (OR: 1.58, 95% CI: 1.44-1.74, p < 0.001). Prosthetic valve complications, on the other hand, showed a consistent decrease, with OR significantly dropping in 2018 (OR: 0.48, 95% CI: 0.33-0.70, p < 0.001) and remaining low through 2020 (OR: 0.55, 95% CI: 0.38-0.80, p = 0.001). Non-traumatic hemopericardium demonstrated a significant rise, with OR increasing from 2019 (OR: 1.61, 95% CI: 1.10-2.34, p = 0.013) to 2020 (OR: 1.72, 95% CI: 1.18-2.51, p = 0.005).

Vascular Complications

Vascular complications, including thoracic vascular injuries, retroperitoneal injuries, and extremity vascular complications, showed no statistically significant differences across the years.

Early readmission analysis

**Figure 2:**
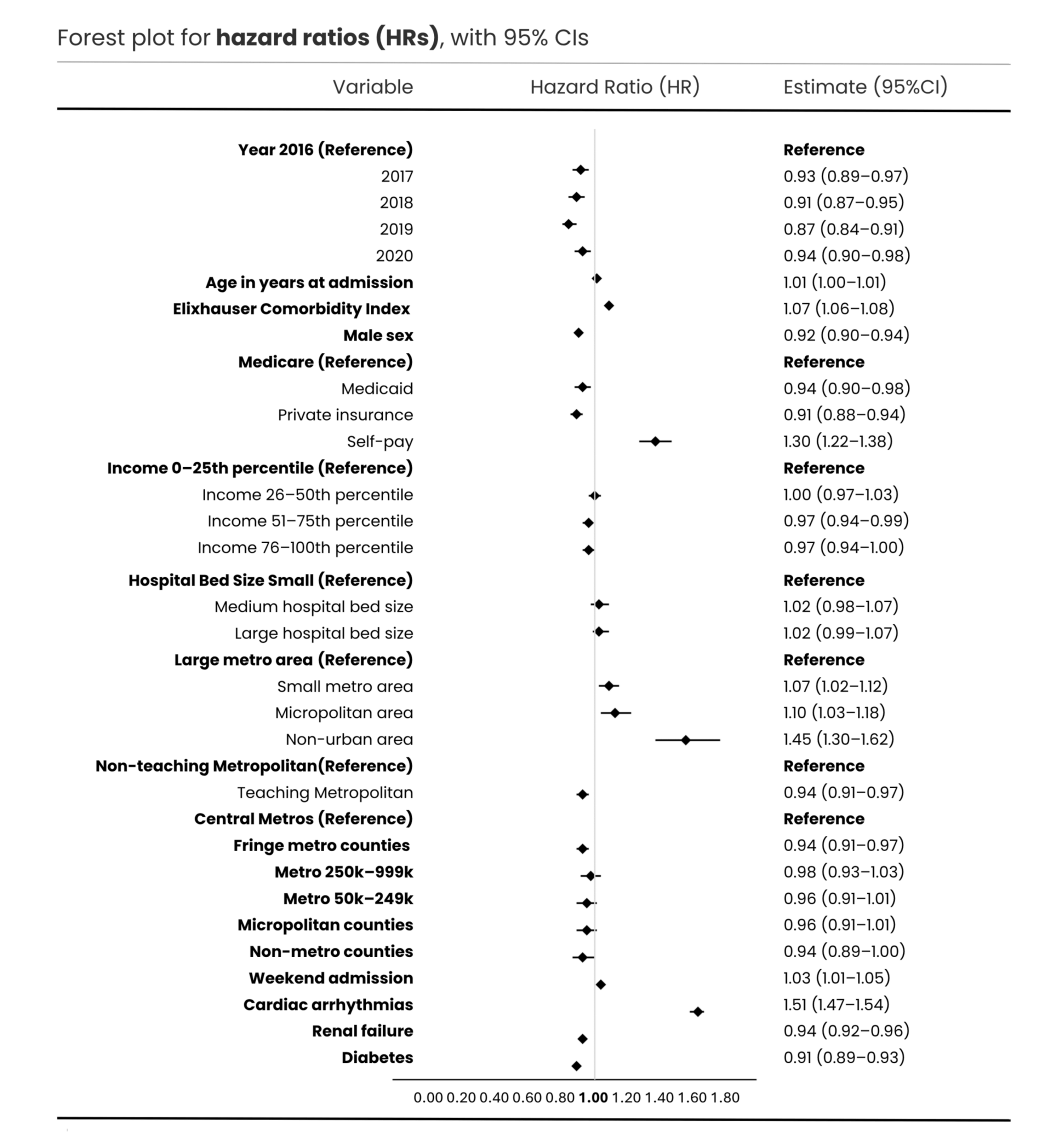
Predictors of 30-day readmission after acute myocardial infarction

In multivariable Cox regression, 30-day readmission risk declined over time, with lower hazards from 2017 to 2019 (HR 0.93 [95% CI 0.89-0.97] to 0.87 [0.84-0.91]), with a modest increase in 2020 (HR 0.94 [0.90-0.98]; all p ≤ 0.002) (Figure 2). Increasing age (HR 1.010 [1.004-1.012]) and comorbidity burden (HR 1.07 [1.06-1.08]) were associated with higher risk, while male sex was protective (HR 0.92 [0.90-0.94]; p < 0.001). Compared with Medicare, Medicaid (HR 0.94 [0.90-0.98]) and private insurance (HR 0.91 [0.88-0.94]) were associated with lower risk, whereas self-pay increased risk (HR 1.30 [1.22-1.38]). Non-metropolitan residence was associated with increased risk (HR up to 1.45 [1.30-1.62]), while metropolitan teaching hospitals were protective (HR 0.94 [0.91-0.97]; p < 0.001). Cardiac arrhythmias markedly increased risk (HR 1.51 [1.47-1.54]), whereas renal failure (HR 0.94 [0.92-0.96]) and diabetes (HR 0.91 [0.89-0.93]) were associated with lower hazards; DNR status showed a strong association with readmission (HR 7.83 [7.62-8.05]; p < 0.001).

Readmission Complications

Procedural complications: Key procedural interventions, including in-hospital CPR, defibrillation, ECMO, and ventilator use, showed no statistically significant changes in odds across the study period, indicating consistent utilization patterns. However, among patients with renal failure admitted for acute MI, the odds of requiring inpatient hemodialysis were markedly elevated (OR: 52.52, 95% CI: 43.75-63.03, p < 0.001), emphasizing the critical need for renal support in this vulnerable population. The use of vasopressors increased significantly over the study period, with odds steadily rising from 2016 (OR: 1.48, 95% CI: 1.02-2.17, p = 0.040) to 2020 (OR: 2.19, 95% CI: 1.51-3.18, p < 0.001), reflecting a greater reliance on circulatory support. In contrast, the likelihood of central line placement significantly decreased, with odds dropping from 2019 (OR: 0.83, 95% CI: 0.74-0.92, p = 0.001) to 2020 (OR: 0.85, 95% CI: 0.75-0.95, p = 0.004), potentially indicating improved procedural efficiencies or the adoption of less invasive management strategies.

Post-procedural complications: The odds of periprocedural circulatory complications significantly decreased over the study period, with significant reductions from 2016 (OR: 0.59, 95% CI: 0.40-0.89, p = 0.012) to 2020 (OR: 0.30, 95% CI: 0.17-0.51, p < 0.001). This trend may indicate improvements in procedural techniques or perioperative care. Periprocedural bleeding also showed significantly reduced odds during readmissions, with odds ratios ranging from 0.40 [95% CI: 0.23-0.68, p = 0.0008] to 0.07 [95% CI: 0.03-0.17, p < 0.001]. The odds of post-procedural anemia significantly increased in 2020 (OR: 1.16, 95% CI: 1.06-1.26, p = 0.001), indicating a rise in anemia cases associated with procedures performed during this period. Post-procedural respiratory complications significantly decreased over time, with odds consistently dropping from 2016 (OR: 0.40, 95% CI: 0.19-0.83, p = 0.014) to 2020 (OR: 0.37, 95% CI: 0.17-0.81, p = 0.013).

Cardiac-specific complications: The odds of non-inflammatory pericardial effusion significantly increased in patients with readmissions over the study period, particularly in 2020 (OR: 1.62, 95% CI: 1.34-1.97, p < 0.001), reflecting a rising trend in this complication. Periprocedural circulatory complications exhibited significantly reduced odds over the years, with the lowest observed in 2019 (OR: 0.26, 95% CI: 0.16-0.43, p < 0.001) and 2020 (OR: 0.30, 95% CI: 0.17-0.51, p < 0.001), suggesting the effectiveness of improved management strategies.

Vascular complications: Vascular complications during readmission had fluctuating odds but did not show consistent statistical significance across the period.

## Discussion

This study evaluates 30-day readmissions following acute MI, a significant contributor to U.S. healthcare costs, which exceed $1 billion annually [19]. In our study, older age, male sex, and higher Elixhauser Comorbidity Index scores were more prevalent among patients who were readmitted. Socioeconomic factors, including Medicaid insurance and low-income status, were associated with an increased risk of readmission. Meanwhile, metropolitan areas and large teaching hospitals showed lower odds of readmissions. These findings align with prior studies by Li et al. [5] and Rachoin et al. [20], highlighting the interplay between clinical and socioeconomic determinants in post-MI readmissions.

Thirty-day readmission rates are declining largely owing to improved care transitions. Post-discharge follow-up by transitional care teams comprising nurses and social workers, along with proactive phone calls within the first 7 days of discharge and routine post-discharge physician appointments, has contributed to this declining trend [21]. The utility of the electronic health record (EHR) secure messaging option has also improved communication between hospital teams and outpatient providers, leading to enhanced collaboration and ensuring quality patient care. However, increased EHR utilization has been linked to a greater cognitive burden and attention-switching demands for physicians. Lew et al. [22] have emphasized its negative impacts.

Previous literature has established that socioeconomic factors, such as low salary, low literacy, and lack of private insurance, affect readmission, primarily due to insufficient coping skills, low health literacy, inadequate living conditions, and limited access to healthcare [23,24]. These findings were consistent in our study. These disparities have long been talked about across different diseases like COPD, asthma [25], heart failure [26], and procedures like hematopoietic cell transplant [27]. The effect of these disparities on post-MI readmission status has not yet been discussed. Having the Centers for Medicare & Medicaid Services (CMS) insurance puts the population at a disadvantage, with limited access to specific procedures and low reimbursement for outpatient services. This not only takes an emotional toll on patients but also imposes a financial burden on US healthcare spending through readmission expenditures. Policymakers need to amend CMS insurance programs to improve healthcare access for the population by expanding the network of safety-net hospitals and increasing the scope of procedures and services eligible for CMS reimbursement.

Kwok et al. (2020) conducted a similar study with an earlier study period (2010-2014), examining unplanned readmission following acute MI. The earlier study comparably demonstrated a decline in early unplanned readmissions, with the rate dropping from 13% in 2010 to 11.5% in 2014. Risk factors for readmission included older age, multiple comorbidities, and female gender [28]. The Hospital Readmissions Reduction Program (HRRP), introduced in 2009, aimed to reduce 30-day readmissions for heart failure, acute MI, and pneumonia by imposing financial penalties of up to 3% on hospitals with above-average risk-standardized readmission rates (RSRRs) [29]. While there was no significant decrease in 30-day readmission mortality following its implementation, a surprising increase in 30-day, 90-day, and 1-year mortality was observed in heart failure patients, as reported by some independent studies [30]. This could be attributed to what some label as ‘gaming of the system’, with reports of increasing observation stays, inappropriate triage protocols, and delaying readmissions beyond the 30th discharge day.

Although overall hospital readmissions have been declining, our analysis revealed an increasing trend in the Elixhauser Comorbidity Index, which rose gradually from 3.8 to 4.0 over four years. Many have argued that tools like the Elixhauser Comorbidity Index can be used to predict readmissions. This could potentially further decrease the readmission numbers that we observed. Rana et al. [31] argued in their study that their 7-factor predictive score, derived from the Elixhauser comorbidities, could help decrease 30-day cardiac-specific hospital readmissions. Still, a systematic review by Smith and Johnson [32] argued that, even though models like these can have modest predictive power, they fail to provide real-time, actionable information to practically reduce hospitalizations, as most predictive models were based on data not readily available at the time of hospitalization. Furthermore, these models also lack generalizability. Keeping that in mind, with all the advancements and improved post-hospital communication between physicians and patients, we anticipate greater practicality for these models moving forward.

Limitations

This study uses the NRD database, which has several limitations. The NRD database is an administrative, claim-based database that uses ICD-9-CM and ICD-10-CM codes for diagnosis and reimbursement, which may vary in detail and accuracy and are subject to misclassification. Validity studies for ICD-9-CM and ICD-10-CM codes are limited. Due to the unavailability of laboratory values in the NRD database, we were unable to assess baseline laboratory results at readmission. As with all studies analyzing data from the NRD database, we were unable to establish causality and could only identify associations. There is also a potential for measured and unmeasured confounding factors not gathered by this database that can influence the findings.

## Conclusions

In conclusion, we noticed declining trends in hospital readmissions in post-MI patients. While these are promising numbers, there is still a need for further interventions to explore the matter in more depth. Multidisciplinary efforts and structured discharge plans have helped reduce the numbers; however, additional efforts are needed to develop readmission-predictor models. Although prior studies failed to demonstrate the practical utility of hospital readmission predictor models, there remains potential for their use. Further, large multi-center trials are needed to validate their applicability and generalizability.
